# The JAX Synteny Browser for mouse-human comparative genomics

**DOI:** 10.1007/s00335-019-09821-4

**Published:** 2019-11-27

**Authors:** Georgi Kolishovski, Anna Lamoureux, Paul Hale, Joel E. Richardson, Jill M. Recla, Omoluyi Adesanya, Al Simons, Govindarajan Kunde-Ramamoorthy, Carol J. Bult

**Affiliations:** 1 The Jackson Laboratory for Mammalian Genomics, Bar Harbor, ME 04609 USA; 2 The Jackson Laboratory for Genomic Medicine, Farmington, CT 06032 USA; 3 Present Address: Institute of Public Health, Washington University of St. Louis, St. Louis, MO 63110 USA

**Keywords:** Conserved synteny, Bioinformatics, Comparative genomics, Candidate gene analysis

## Abstract

Visualizing regions of conserved synteny between two genomes is supported by numerous software applications. However, none of the current applications allow researchers to select genome features to display or highlight in blocks of synteny based on the annotated biological properties of the features (e.g., type, function, and/or phenotype association). To address this usability gap, we developed an interactive web-based conserved synteny browser, The Jackson Laboratory (JAX) Synteny Browser. The browser allows researchers to highlight or selectively display genome features in the reference and/or the comparison genome according to the biological attributes of the features. Although the current implementation for the browser is limited to the reference genomes for the laboratory mouse and human, the software platform is intentionally genome agnostic. The JAX Synteny Browser software can be deployed for any two genomes where genome coordinates for syntenic blocks are defined and for which biological attributes of the features in one or both genomes are available in widely used standard bioinformatics file formats. The JAX Synteny Browser is available at: http://syntenybrowser.jax.org/. The code base is available from GitHub: https://github.com/TheJacksonLaboratory/syntenybrowser and is distributed under the Creative Commons Attribution license (CC BY).

**Electronic supplementary material:**

The online version of this article (10.1007/s00335-019-09821-4) contains supplementary material, which is available to authorized users.

## Introduction

Conserved synteny describes a condition in which shared ancestry is reflected as similar genome feature content and order along a chromosome in different species. Visualizing and navigating between regions of conserved synteny is a common task in studies where biological annotations and genome features in one species are used to prioritize candidate genes for a complex trait mapped in another species. For example, a researcher might start with the genome location of a trait mapped in a Genome Wide Association Study (GWAS) or linkage study in humans and use the conserved syntenic location in the mouse or rat genomes to identify candidate genes underlying the trait of interest (Berisha and Smith 2011; Mell et al. 2019; Stoll et al. 2000; Sugiyama et al. 2001). Comparing regions of conserved synteny across organisms is also used extensively for investigations into the processes underlying genome evolution (Nakatani and McLysaght 2017).

Many software tools have been developed for computing and/or visualizing conserved syntenic regions for multiple genomes (Baek et al. 2016; Catchen et al. 2009; Drillon et al. 2014; Haug-Baltzell et al. 2017; Lee et al. 2016; Lyons et al. 2008; McKay et al. 2010; Meyer et al. 2009; Pan et al. 2005; Revanna et al. 2012; Riley et al. 2012; Sinha and Meller 2007; Soderlund et al. 2011; Xu et al. 2016). However, existing user interfaces are limited to the visualization of the regions of conserved synteny and to the genome features contained within the syntenic blocks; existing software tools do not support that ability of researchers to search for and highlight genome features within syntenic blocks according to the functional and phenotypic attributes associated with the genome features. To address this usability gap, we have developed and implemented the JAX Synteny Browser.

The Synteny Browser allows users to search for and selectively display genome features within syntenic blocks according to the biological and functional annotations associated with the features. For example, after a researcher identifies a conserved syntenic block of interest, they can subsequently selectively display genome features in the Reference and/or Comparison genome by *feature type* (protein-coding, long non-coding RNA, micro RNA, etc.), *function* (Gene Ontology annotation), *disease association* (Disease Ontology annotation), and/or *abnormal phenotype* (Mammalian Phenotype Ontology annotation).

The current implementation of the JAX Synteny Browser supports mouse and human genomes; however, the software design is genome agnostic and can be applied to any two genomes for which relevant annotation data in commonly used data formats exist. For the implementation described here, the genome features and biological annotations for the laboratory mouse come from the Mouse Genome Informatics (MGI) database (Bult et al. 2018); corresponding data for the human genome comes from multiple sources, including NCBI’s Gene (Brown et al. 2015), the Disease Ontology (Schriml et al. 2019), and the Gene Ontology (GO) Consortium (The Gene Ontology 2019). A summary of the resources is provided in Online Resource 3.

## Materials and methods

### Software

The JAX Synteny Browser is a web-based synteny viewer application preloaded with human and mouse data. Data from different public sources were extracted and processed using custom Python-based ETL (extract, transform, load) scripts and loaded into an SQLite (https://www.sqlite.org/) database. The data are accessed through an Application Programming Interface (API) developed with the Flask (http://flask.pocoo.org/) microframework for Python 3. The front-end user interface utilizes the API endpoints to get data in JavaScript Object Notation (JSON) format to search, filter and to generate the synteny visualizations. Users can access the public version of the Browser or download the source code and install a version locally from a Docker container to load and visualize private data. A graphical overview of the JAX Synteny Browser system architecture is provided in Online Resource 1. The code base is available from GitHub: https://github.com/TheJacksonLaboratory/syntenybrowser and is distributed under the Creative Commons Attribution license (CC BY).

### Data sources and processing

The JAX Synteny Browser relies on data that are available in standard data formats such as General Feature Format (GFF3) for genome features, Gene Association Format (GAF2) for biological annotations and Open Biological and Biomedical Ontology (OBO) for ontologies. The full list of data types, file formats, and data sources used for the implementation of the synteny viewer described here are provided in Supplementary materials (Online Resource 3). Four specialized Data Loader scripts (written in Python) are used to extract, transform, and load all necessary biological data for the application. A single shell script is available to run the four Data Loaders, which sequentially load features and syntenic block data, ontologies, and biological annotations in the database.

Any method for defining synteny blocks can be used as long as the data are represented in the format described in the software documentation. For the current implementation of the browser for mouse and human, regions of synteny were generated using approaches described by Nadeau and Taylor (1984) and Ghiurcata and Moret (2014) for defining framed, collinear syntenic blocks. The starting point for the algorithm is a set of genes and their genome locations from both organisms and a set of orthology relationships represented as a binary relation of gene pairs. Starting with genome A, the genes are sorted by chromosome and start position, and numbered starting at 1. The same operation is then performed for genome B, sorting and numbering its genes. Next, scanning the genes of A in order, the succession of indices of the orthologs in B are noted. Each maximal run of sequential indices, in either ascending or descending order, defined one synteny block. The boundaries of the block in each genome are defined by the outer limits of the genes involved. The block’s orientation is “ + ” if the B indices increased and “−” (an inversion) if they decreased.

The format for describing blocks of conserved synteny is a simple tab-delimited format in which each line contains the first organism’s chromosome, taxon ID, start, and end of the syntenic block followed by the second organism’s chromosome, taxon ID, start, and end. The last two columns of the file must include the orientation of the block (±) and an identifier for the block. The only requirement for block identifiers is that they must be unique across the file. An example of the format is as follows:

$$1\; 10090\; 320590 1\; 9299878\; 8\; 9606\; 49909789\; 55526155\; {\text{-}}\; {\text{ID}}{=}{\text{SynBlock}}{:}{\text{mmhs}}{:}1$$

### Database and configuration files

The data available in the JAX Synteny Browser are stored and extracted from an SQLite database and are updated quarterly. Two user-defined configuration files, one for each species’ genome (reference and comparison), provide specific application level settings and meta-data such as the ontologies and genome features available for interaction. The configuration files are separate from the database itself and are formatted as specified in the software documentation available from the Browser web site (see also, Online Resource 2).

### Application layer and application programming interface (API)

The application layer exposes the data available for display as a microservice. The microservice is callable from external web clients and provides multiple endpoints, each encoded as a uniform resource identifier (URI). Each endpoint gets arguments from the client to narrow queries and build specific structured data objects optimized for the client. These data objects are returned to the client in JSON format.

### User interface and visualization

The user interface and interactive visualizations are built with a variety of open source technologies, namely D3 (https://d3js.org/), jQuery (https://jquery.com/), and Twitter Bootstrap (https://getbootstrap.com/. API microservice requests are sent via AJAX and the returned data objects are processed using JavaScript and D3 to generate Scalable Vector Graphics (SVG) markup. Due to the large size of the data and the resulting number of SVG elements displayed on the client’s side, the visualization code has been optimized to reduce Random Access Memory (RAM) consumption and improve the speed of rendering.

## Results

**Fig. 1:**
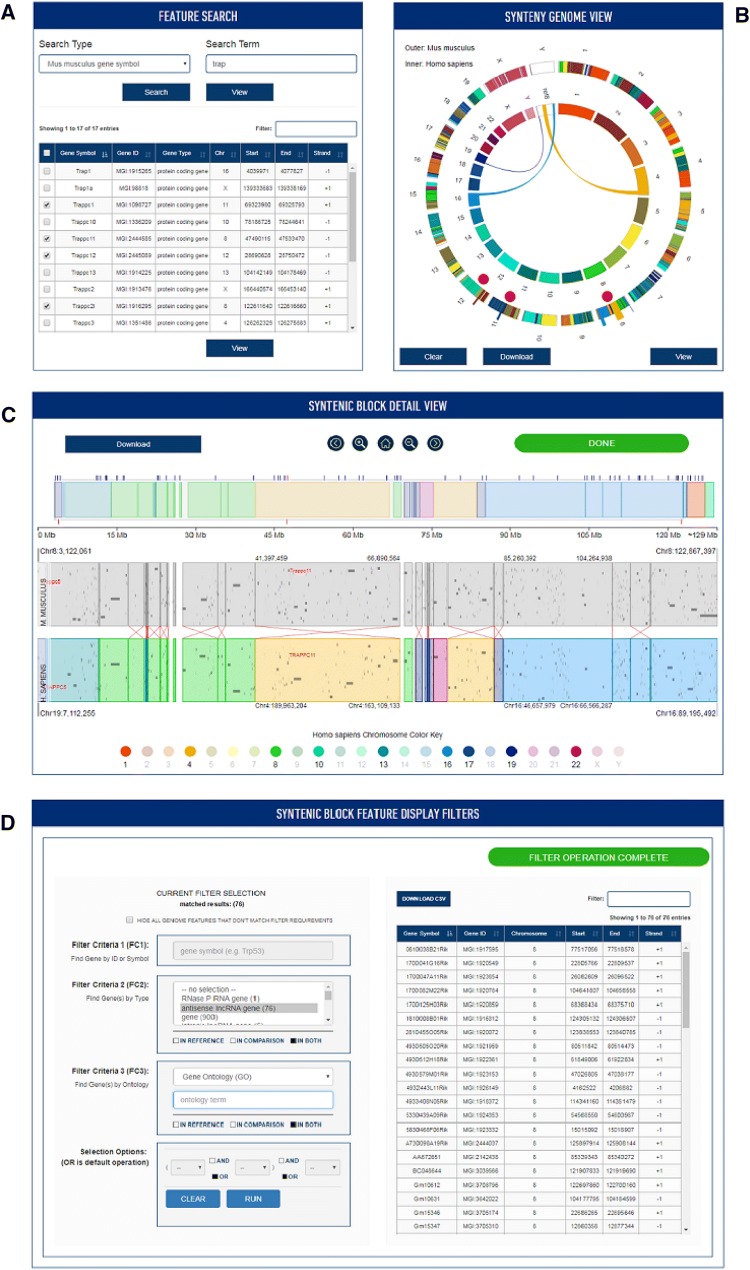
The JAX Synteny Browser is organized as four main panels: **a** the *Feature Search* panel allows users to search the reference species by genome features of interest or mapped phenotypes. **b** The *Genome View* panel presents a circos plot to display a genome level view for both the reference genome (outer ring) and comparison genome (inner ring). **c** The *Syntenic Block Detail View* allows users to browse and focus on features in both the reference (top track) and comparison (bottom track) species at the chromosome and block levels. **d** The *Syntenic Block Feature Display Filters* panel allows users to search for and highlight genome features based on different biological attributes such as feature type and function. Genome features that match the search criteria can be displayed selectively

The JAX Synteny Browser (http://syntenybrowser.jax.org/) is available as a web-based application with a user interface consisting of four panels: Feature Search, Synteny Genome View, Syntenic Block Detail View, and Syntenic Block Feature Display Filters (Fig. 1). Drop down menus in the Syntenic Genome View panel allow users to switch the reference and comparison genomes.

### Feature search

The Feature Search panel (Fig. 1a) allows users to search for genome features in the Reference genome by feature symbol or annotation term. By default, the Reference genome is the mouse. The Reference genome can be changed using the Settings menu, which is indicated by a cog icon in the top right hand corner of the interface.

From the search results, users can select some or all of the data elements for viewing in the Synteny Genome View panel (Fig. 1b). Alternatively, users can skip the Feature Search panel and begin their exploration of conserved synteny by selecting one of the reference chromosomes displayed in the outer ring in the Synteny Genome View panel. This action will generate color arcs showing the regions of conserved synteny in the comparison genome.

### Synteny genome view

The Synteny Genome View (Fig. 1b) employs a circular display with the reference genome as the outer ring and the comparison genome in the inner ring. Selected data elements resulting from a Feature Search are indicated by the red dots along the reference genome; the locations of the selected data elements are also elevated slightly for emphasis. When a user clicks on a red dot, the selected region is displayed in the center ring and colored arcs show the location of the syntenic regions in the comparison species. When a user clicks on one of the indicator dots, the location of the syntenic blocks are shown in the Genome View panel and simultaneously the associated regions are displayed in the Syntenic Block detail View.

### Syntenic block detail view

The Syntenic Block Detail panel (Fig. 1c) displays the genome features in the reference and comparison species. The reference genome is shaded in gray and the comparison genome is color coded to highlight the chromosomal locations of the conserved syntenic blocks. Navigation operations in Block View include zoom and pan. Guidelines to indicate the anchor features used to define blocks can be toggled on and off.

### Syntenic block feature display filters

Existing synteny browsers support the display of conserved synteny between two or more genomes and also the display genome features within the blocks. A unique function of the JAX Synteny Browser lies in the Syntenic Block Feature Display Filters. The filter interface allows researchers to search for and selectively display genome features within the region of conserved synteny that match biological attributes of interest (Fig. 1d).

Three search criteria are supported: genome feature name or symbol, feature type, and annotation term. Search criteria can be combined using Boolean operators. The searches can be limited to the reference or comparison genomes, or set to include matches from either genome. A table of matching features is displayed and can be downloaded as a comma separated file. The locations of matching features are highlighted in the Block View using vertical tick marks (Fig. 1c). An optional toggle allows users to exclude from the display any features that do not match the search criteria. To support access to detailed information available for the genes displayed in the Syntenic Block Detail View window hyperlinks to external data resources are provided: NCBI Gene for human genes and MGI for mouse genes.

## Discussion

The Jackson Laboratory (JAX) Synteny Browser is an interactive web-based genome browser for conserved synteny. The browser allows researchers to highlight or selectively display genome features in the Reference and/or the Comparison genome according to the biological attributes of the features. The basic workflow for using the Browser involves four steps:

1. selecting the Reference genome,
2. specifying a genome region of interest on the Reference,
3. visualizing the region of interest and its corresponding conserved syntenic block(s) in the Comparison genome, and
4. selectively highlighting genes in the Reference and Comparison genomes based on their biological attributes.

Two use cases are described below give examples of specific uses of the Synteny Browser. An illustrated guide to these use cases is available from the Documents menu on the Synteny Browser web site.

### Identifying candidate genes in a mapped interval for human lung cancer susceptibility

A region of human chromosome 6 (6q23–25; GRCm38 chr6:130300000–161000000 bp) was identified previously as a linkage interval associated with human lung cancer susceptibility (Bailey-Wilson et al. 2004). Because this linkage interval overlapped regions of allelic loss observed in several different types of cancer, the authors hypothesized that genes involved in regulating apoptosis (tumor suppressor genes) would be likely candidates for the susceptibility phenotype.

To use the JAX Synteny Browser to find potential candidate genes for the human lung cancer susceptibility locus a researcher would first select ‘human’ as the Reference genome using the Settings menu. Next, they would navigate to the region of interest on human chromosome 6 using one of two options. For the first option, human chromosome 6 could be selected in the Synteny Genome View graphic. Clicking on the View button in this panel results in the entire chromosome being displayed in the Syntenic Block Detail window. The display interval would then be refined to a region of interest using the slider on the chromosome overview graphic. Alternatively, the coordinates of a genomic interval for the Reference genome could be entered in the dialog box within the Settings menu. After the “Update View” button is clicked, the user-specified genomic region would be displayed in the Syntenic Block Detail panel.

**Fig. 2:**
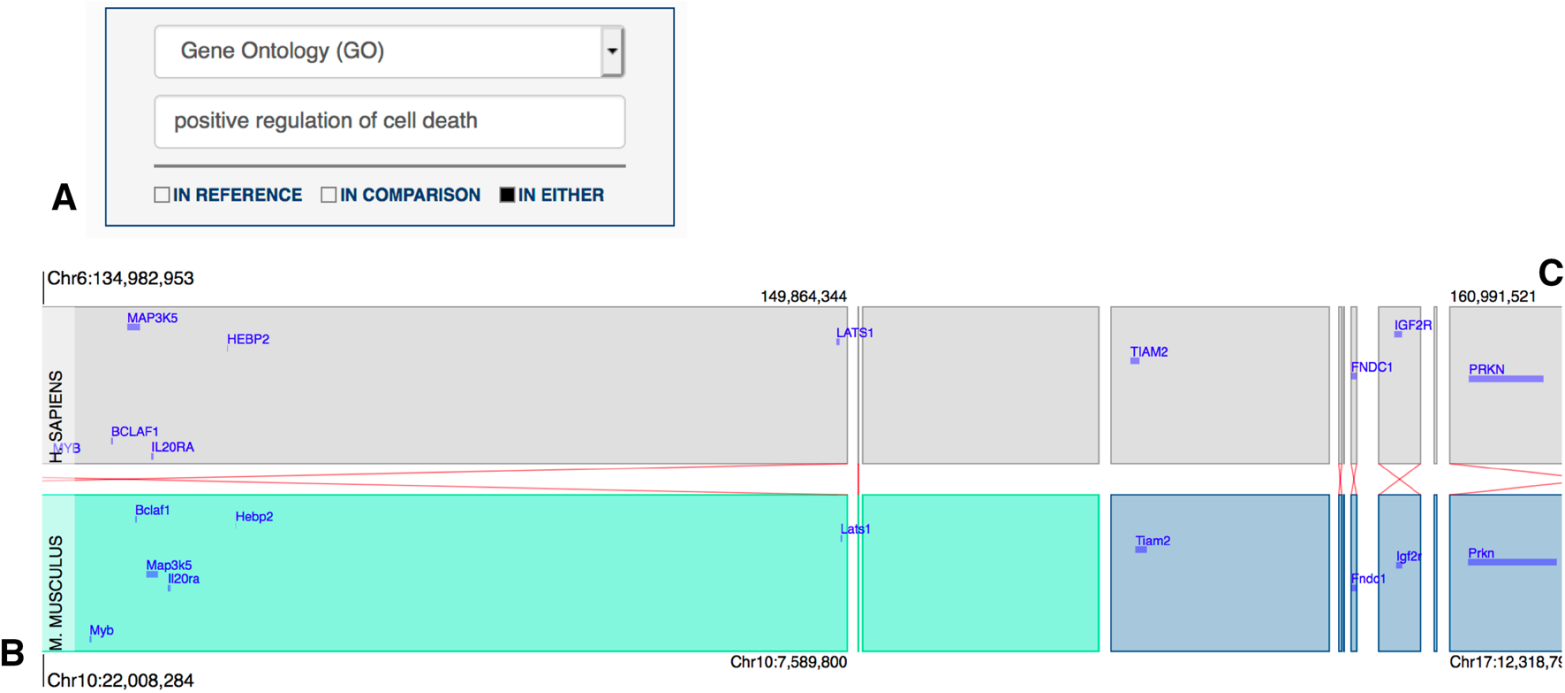
Screenshot of the *Syntenic Block Detail View* for genes annotated to the GO term, positive regulation of cell death: **a** the dialog box in the *Syntenic Block Feature Display Filters* panel set to search both genomes for genes annotated to the GO term of interest. **b** Search results displayed in the *Syntenic Block Detail View* using the option to turn off all genome features except for features that match the search criteria. The red lines indicate that the regions of synteny are in opposite orientations in the mouse and human genomes. They are displayed in the same orientation to simply the interface. This option can be turned off in the *Settings* menu

Once the extent of the genome region displayed in the Syntenic Block Detail View window was finalized, a researcher could search for genome features according to their biological and functional annotations med using the Syntenic Block Features Display Filters function. For the lung cancer susceptibility interval, a search for genome features that are annotated to the GO biological process term “positive regulation of cell death” in *either* the Reference or Comparison genome resulted in eleven genes (MYB, *CCN2, MAP3K5, HEBP2, BCLAF1, TIAM2, IL20RA, LATS1, FNDC1, IGF2R, and PRKN*) being highlighted in the Syntenic Block Detail View (Fig. 2). One of these genes, *PRKN*, is returned because of annotations for the mouse gene only; *MYB* and *TIAM2* are returned based on human gene annotations only. Four of the genes (*CCN2, IL20RA, IGF2R, and PRKN*) were identified by the authors of the mapping paper as likely candidate genes. The ability for researchers to explore biological annotations of the genome features for one organism or for both is one of the special features of the JAX Synteny Browser.

### Identifying candidate genes for Type 2 diabetes QTL in mice

The Quantitative Trait Locus (QTL) *T2dm2sa* (type 2 diabetes mellitus 2 in SMXA RI mice) was identified as a region of mouse chromosome 2 associated with impaired glucose tolerance, hyperinsulinemia, and high body mass index (BMI) (Kobayashi et al. 2006). To identify possible candidate genes in the QTL interval using prior biological knowledge about the genome features in this chromosomal region, a user would first use the Feature Search option to search for the *T2dm2sa* QTL (GRCm28; Chr2:29417935-148533014) from the Mouse Genome Informatics (MGI) database. Selecting *T2dm2sa* from the Feature Search results table and then clicking on the View button locates the features on mouse chromosome 2 in the Synteny Genome View panel. Clicking on chromosome 2 and then the View button from this panel generates the Syntenic Block Detail panel.

To explore annotated functions and phenotype associations of mouse genes within and around the QTL region the researcher could limit the annotation searches to the mouse (Reference) genome and then use the Syntenic Block Features Display Filters tool to find genes annotated to relevant phenotype terms from the Mammalian Phenotype (MP) ontology (Smith and Eppig 2012). A search for the MP term, impaired glucose tolerance, identifies thirteen genes that fall within the boundaries of the *T2dm2sa* QTL interval: *Pkn3, Lcn2, Dpm2, Zbtb43, Bbs5, Commd9, Hipk3, Pax6, Hdc, Ap4e1,Chgb, and Pcsk2*. A search for the MP term, increased circulating insulin level (hyperinsulinemia), returns seven genes: *Lcn2, Slc2a8, Dpp4, Bdnf, Hdc, Snap25, and Pcsk2.* The *Snap25* gene is also returned from a search for the phenotype term, increased body mass index. The candidate gene suggested by the authors for the *T2dm2sa* QTL was *Nr1h3* (nuclear receptor subfamily 1, group H, member 3), a transcription activity regulator with a well-documented role in lipid homeostasis according to GO annotations available from MGI (http://www.informatics.jax.org/go/marker/MGI:1352462). The suggested association of this gene with the *T2dm2sa* was based on the analysis of genes differentially expressed in mice with targeted mutations in *Nr1h3* compared to wild type mice (Stulnig et al. 2002); however, the insulin levels and glucose tolerance in *Nr1h3* knockout mice evaluated by the International Mouse Phenotyping Consortium (IMPC) (Munoz-Fuentes et al. 2018) are within normal levels (https://www.mousephenotype.org/data/genes/MGI:1352462) suggesting that *Nr1h3* may not be directly responsible for the phenotypes observed by Kobayashi et al. (2006).

**Fig. 3:**
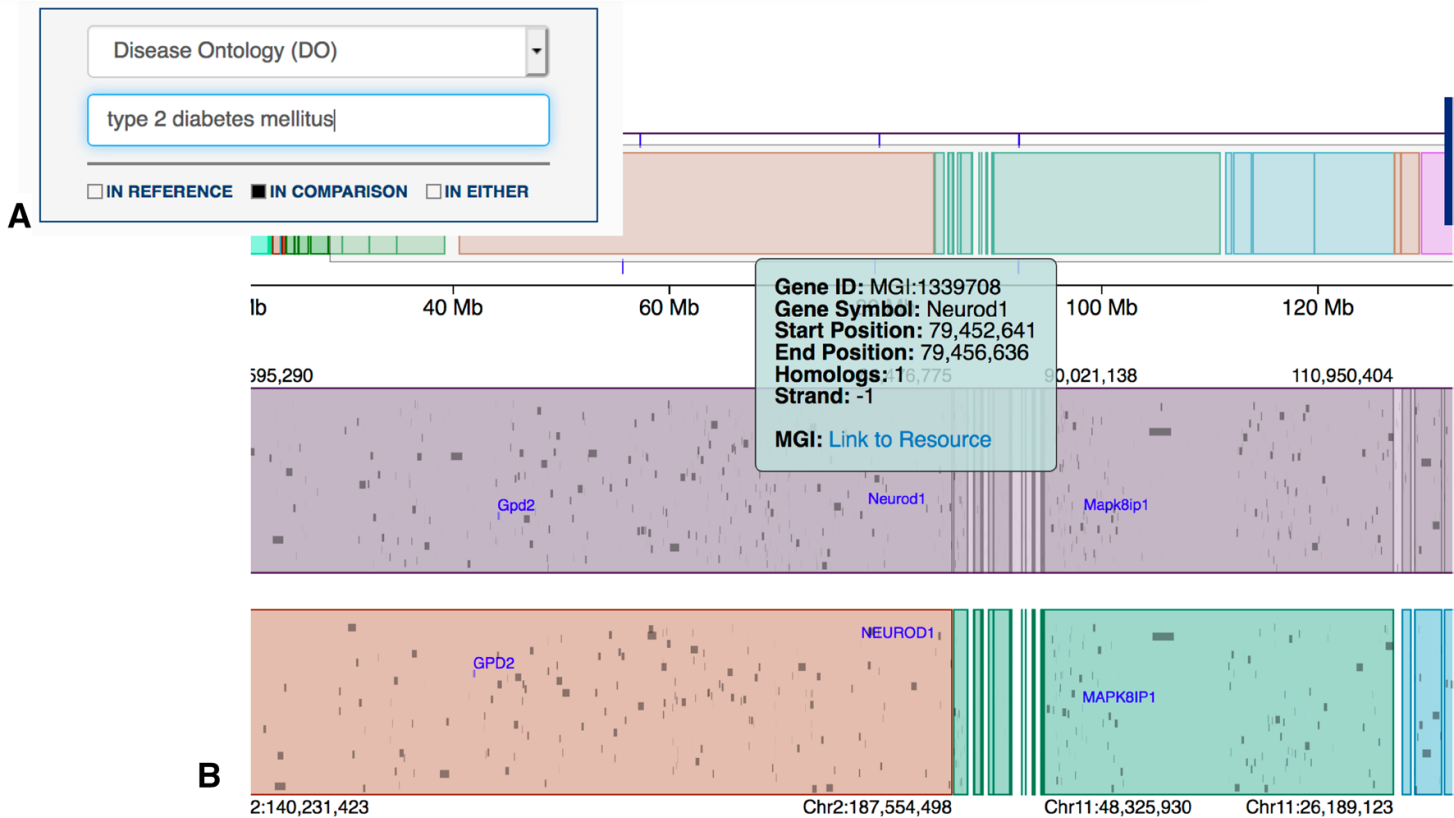
Screenshot of the *Syntenic Block Detail View* for genes annotated to the DO term, type 2 diabetes mellitus: **a** the dialog box in the *Syntenic Block Feature Display Filters* panel with settings to search only the human genome for the DO term of interest. **b** Results displayed in the syntenic block region. The graphic at the top is the overview of the Reference genome (mouse, in this case). The colors correspond to the locations of the regions of synteny in the human genome. The black horizontal line indicates the chromosomal region corresponding to the syntenic block details displayed. The vertical tick marks indicate the locations of the genome features that matched the user’s search criteria shown in panel (**a**). Selecting a genome feature opens a dialog box with links to external resources for more information (MGI for mouse; NCBI Gene for human)

MP terms are relevant only for mouse genes. To identify mouse orthologs of human genes relevant to type 2 diabetes phenotypes, a researcher could limit annotation searches to the human (Comparison) genome and then search for genes annotated to the Disease Ontology term, type 2 diabetes mellitus. This search returns three genes in the human syntenic genome interval for mouse *T2dm2sa*: *GPD2*, *NEUROD1*, and *MAPK8IP1* (Fig. 3). According to the annotation in MGI, the mouse orthologs of *these* genes are associated with phenotypes relevant to type 2 diabetes, but not specifically to the phenotypes of impaired glucose tolerance, hyperinsulinemia, or high BMI. In mice, loss of *Gpd2* is associated with decreased insulin secretion (Eto et al. 1999); loss of Neurod1 is associated with hyperglycemia (Naya et al. 1997); loss of *Mapk8ip1* is associated with abnormal gluconeogenesis and increased insulin sensitivity (Morel et al. 2010).

### Future directions

While annotations of gene function and phenotype/disease associations are one type of information that is useful for the identification of candidate genes associated with complex traits, other key data types (tissue appropriate gene expression and patterns of genome variation, for example) also need to be considered (see Abiola et al. 2003; Flaherty et al. 2005). Thus, although the Synteny Browser offers unique functionality in its current implementation, several extensions are planned for future versions of the software to enhance its utility, including the following:

- The ability to search for and display additional types of genome data (e.g., gene expression, repeat densities, regulatory features, SNPs, etc.)
- Availability of the rat genome feature annotations and conserved synteny with mouse and human genomes
- Feature Searches by gene or QTL name in addition to symbol
- Support for searches by chromosome band (i.e., human 6q23–25)
- Ability for users to switch between different methods for defining conserved syntenic blocks
- The addition of the Human Phenotype Ontology terms to the feature display filter
- Visualization of results from different annotation searches at one time in the Syntenic Block Detail panel

## Electronic supplementary material

Below is the link to the electronic supplementary material.

Supplementary material 1 (DOCX 400 kb)

Supplementary material 2 (PDF 3355 kb)
