# Supplementary material for: The JAX Synteny Browser for mouse-human comparative genomics
Source: Mamm Genome. 2019 Nov 27;30(11):353–61. doi: 10.1007/s00335-019-09821-4 (PMC6892358; doi:10.1007/s00335-019-09821-4)
Supplement: Supplementary file 1 — Supplementary material 1 (DOCX 400 kb) [file 335_2019_9821_MOESM1_ESM.docx]

**Supplementary material**

OnlineResource1.png

Online Resource 1: Graphical overview of the JAX Synteny Browser application architecture


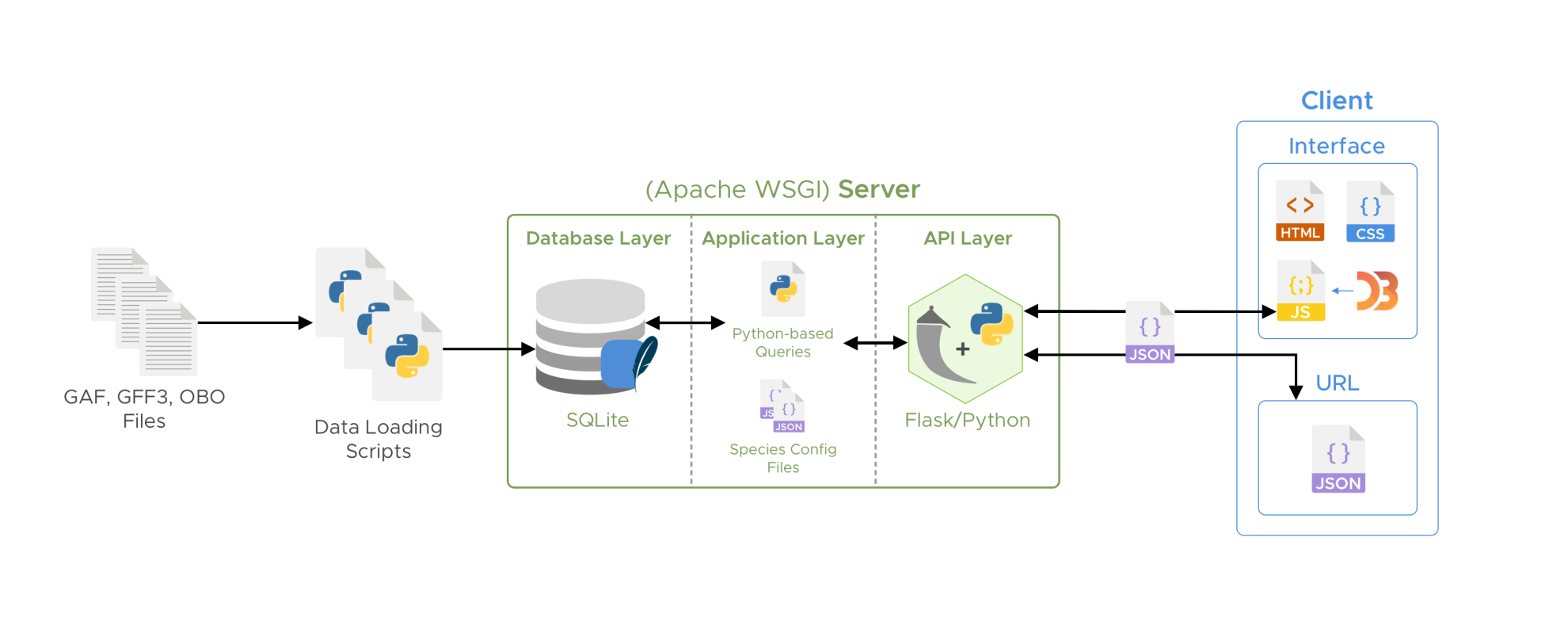


OnlineResource2.png

Online Resource 2: Example of a JSON-encoded application configuration file for the human genome


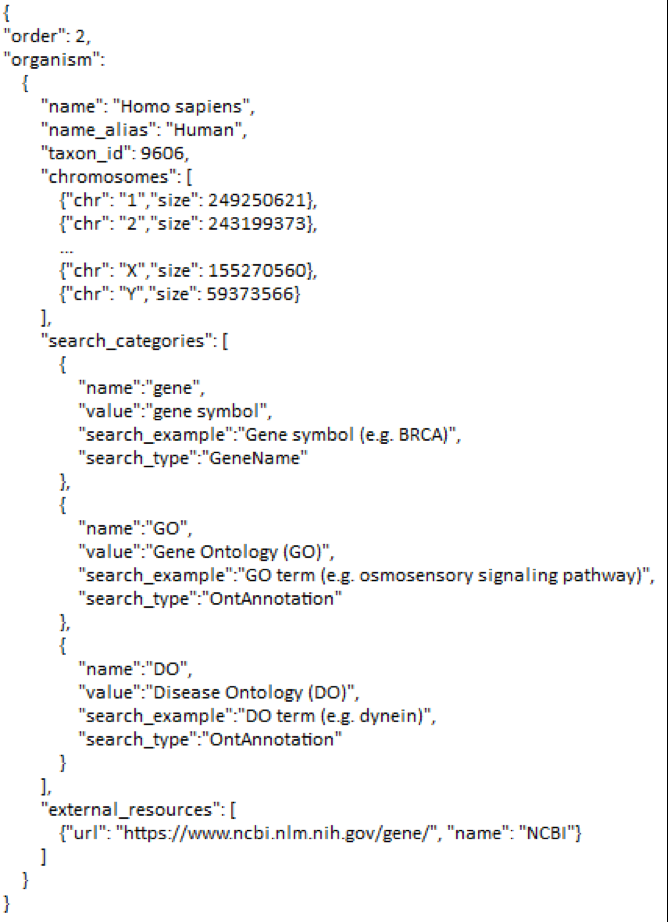


OnlineResource3.pdf

Online Resource 3. Data types, file formats, and data sources for the JAX Synteny Browser
