# Supplementary material for: The JAX Synteny Browser for mouse-human comparative genomics
Source: Mamm Genome. 2019 Nov 27;30(11):353–61. doi: 10.1007/s00335-019-09821-4 (PMC6892358; doi:10.1007/s00335-019-09821-4)
Supplement: Supplementary file 2 — Supplementary material 2 (PDF 3355 kb) [file 335_2019_9821_MOESM2_ESM.pdf]

JAX Synteny Browser Use Cases

http://syntenybrowser.jax.org/

Last revised: November 2019

The basic workflow for using the JAX Synteny Browser involves four steps:
(1) selecting the Reference genome,
(2) specifying a region of interest on the Reference,
(3) visualizing the region of interest and its corresponding conserved syntenic block(s) in the Comparison genome, and
(4) selectively highlighting genes in the Reference and Comparison genomes based on their biological attributes.

Two use cases described below serve to demonstrate this workflow. As the user interfaces for the JAX Synteny Browser evolve with software updates, this document illustrating different use cases will be updated. The most recent version will be available here: http://syntenybrowser.jax.org/docs.html

Use Case #1: Identify candidate genes in a mapped interval for human lung cancer susceptibility

A region of human chromosome 6 (6q23–25; GRCm38 chr6: 130300000-161000000 bp) was identified previously as a linkage interval associated with human lung cancer susceptibility (Bailey-Wilson, et al., 2004). Because this linkage interval also overlaps regions of allelic loss observed in several cancers, the authors hypothesized that genes involved in regulating apoptosis would be good candidates for the susceptibility phenotype.

The first step in using the JAX Synteny Browser to find potential candidate genes for the lung cancer susceptibility locus is to select human as the Reference genome.

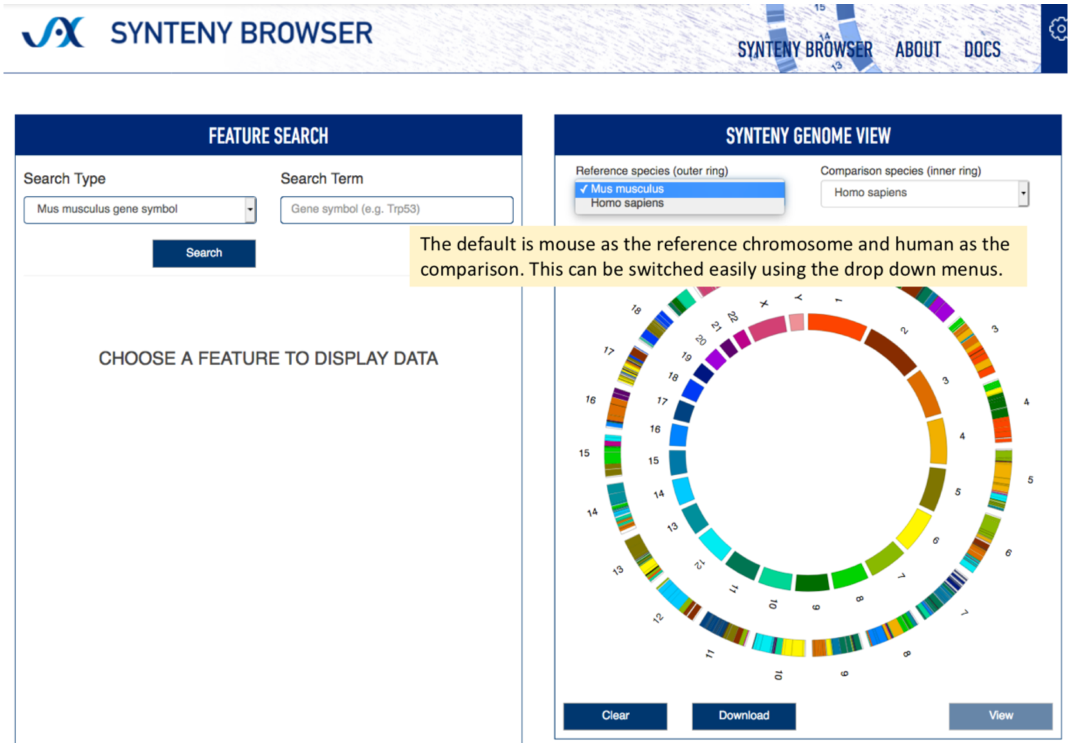

Next, the user navigates to the region of interest on chromosome 6 using one of two options. For the first option, human chromosome 6 is selected in the **Synteny Genome View** graphic. Clicking on the View button in this panel results in the entire chromosome being displayed in the **Syntenic Block Detail** window. The display interval can then be refined interactively using the slider on the chromosome overview graphic. Alternatively, the coordinates of a genomic interval for the Reference genome can be entered in the appropriate dialog box within the **Settings** menu. When the “Update View” button is selected, the user-specified genomic region is then displayed in the **Syntenic Block Detail** panel.

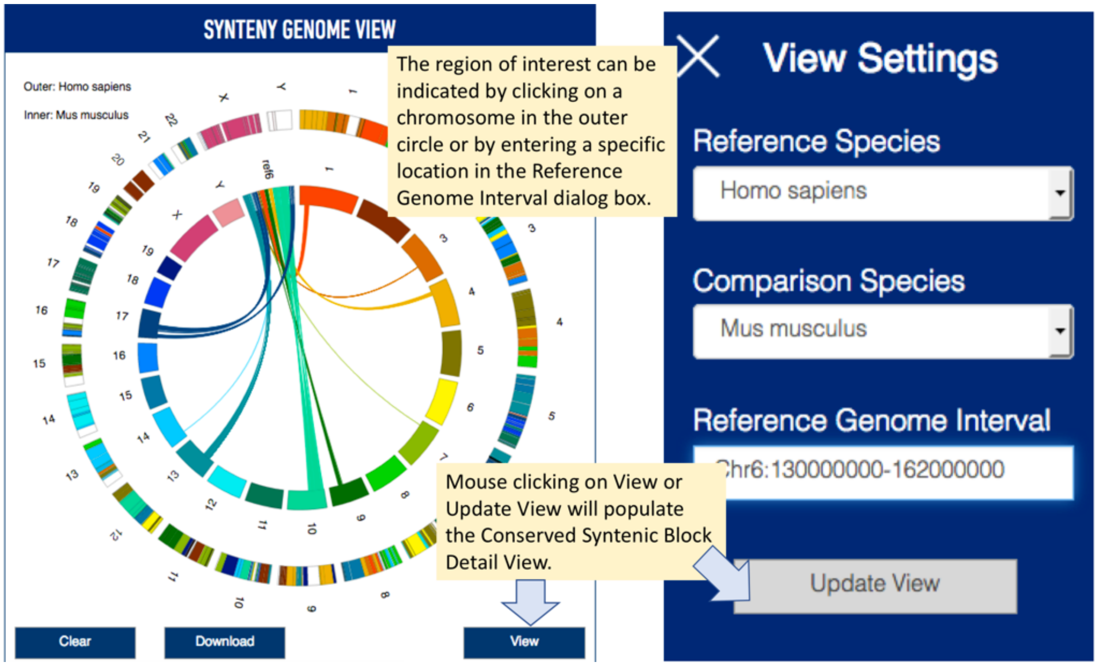

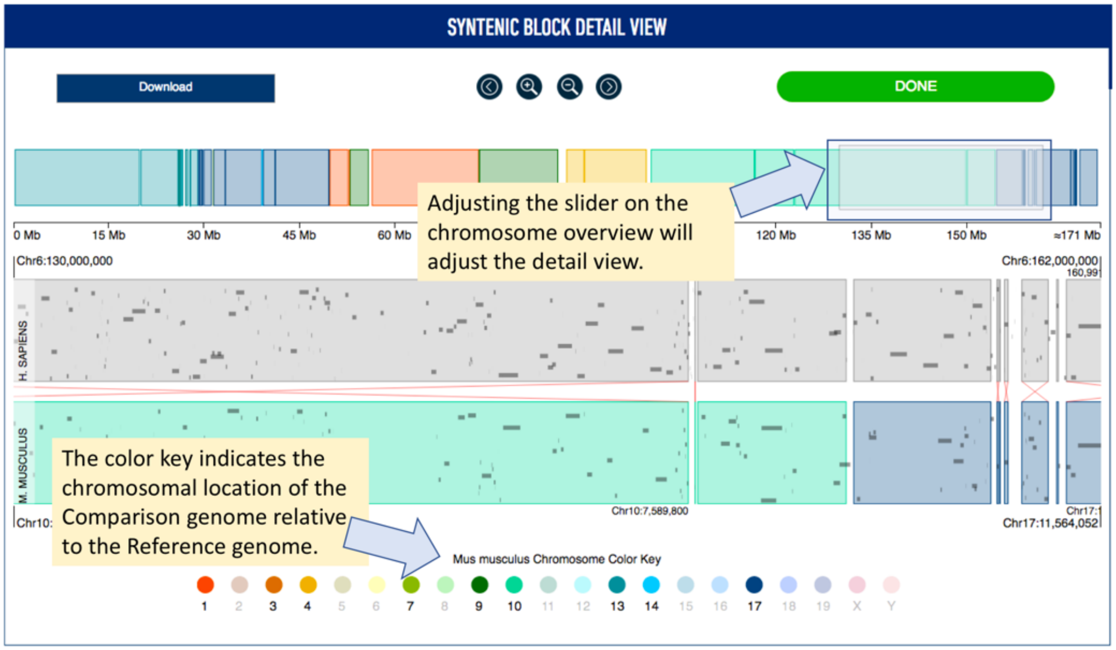

Once the **Syntenic Block Detail View** display is finalized, searches for genome features according to their biological and functional annotations can be performed using the **Syntenic Block Features Display Filters** function. For the lung cancer susceptibility interval, a search for genome features that are annotated to the GO function term of “positive regulation of cell death” in **either** genome results in eight genes (*CCN2, MAP3K5, BCLAF1, IL20RA, LATS1, FNDC1, IGR2R, and PRKN*) being highlighted in the Syntenic Block Detail View. Four of these genes (*IGR2R, PRKN, CCN2, and IL20RA*) were identified by the authors of the mapping paper as likely candidate genes. Clicking on a genome feature opens a dialog box with links to external resources with detailed annotations about the feature (MGI for mouse genes; NCBI for human genes).

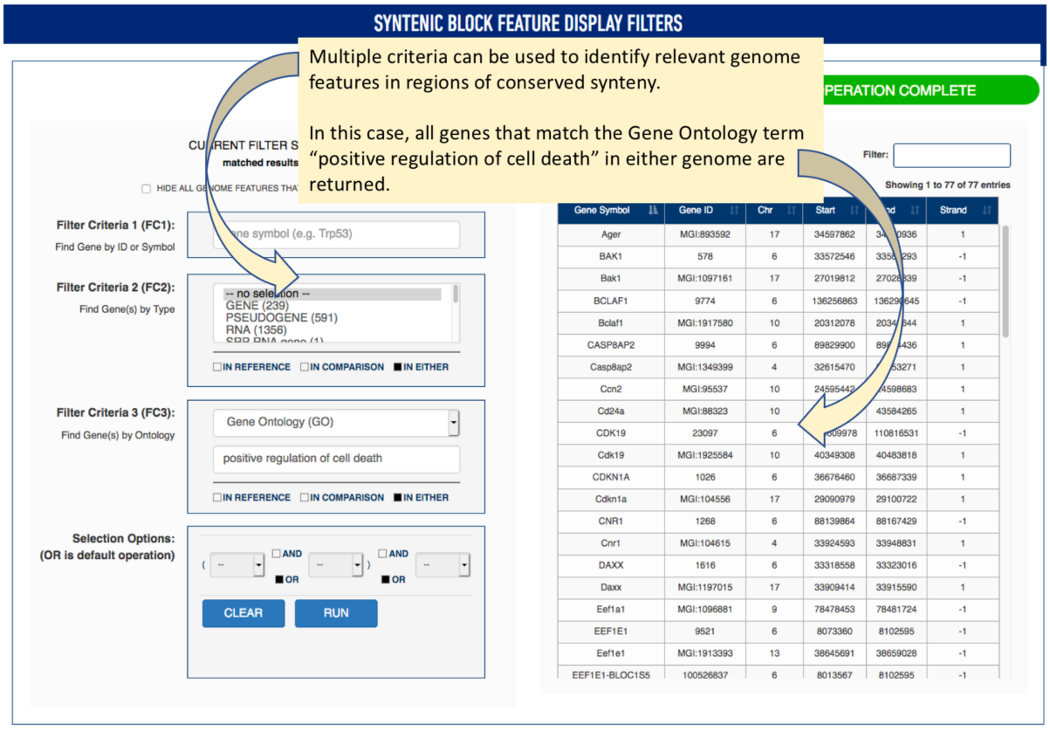

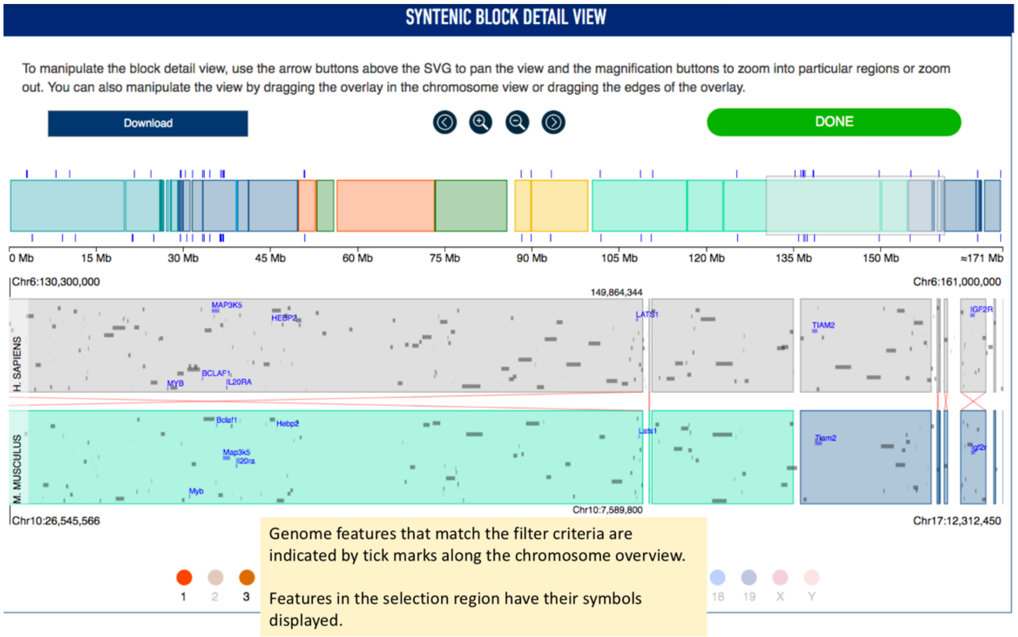

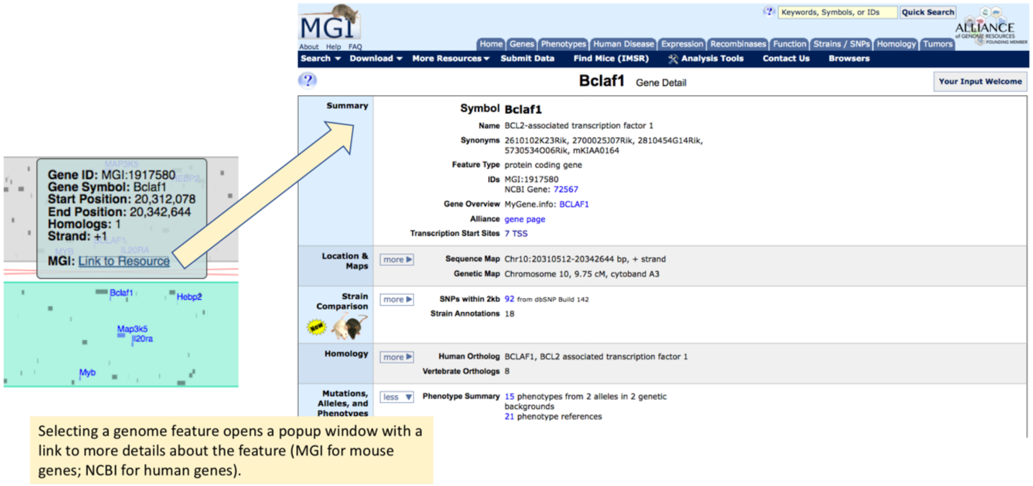

Use Case #2: Identifying candidate genes for Type 2 diabetes

The Quantitative Trait Locus (QTL) *T2dm2sa* (type 2 diabetes mellitus 2 in SMXA RI mice) was identified as a region of mouse chromosome 2 associated with impaired glucose tolerance, hyperinsulinemia, and high body mass index (BMI) (Kobayashi et al. 2006). To identify possible candidate genes in the QTL interval using prior biological knowledge about the genome features in this chromosomal region, a user would first use the **Feature Search** option to search for the *T2dm2sa* QTL (GRCm28; Chr2:29417935-148533014) from the Mouse Genome Informatics (MGI) database.

Selecting *T2dm2sa* from the **Feature Search** results table and then clicking on the View button activates the display of the locus on mouse chromosome 2 in the **Synteny Genome View** panel. Clicking on chromosome 2 (or the red ball) shows the location of the conserved syntenic blocks in the comparison genome. Clicking on the View button from this window activates the **Syntenic Block Detail** panel.

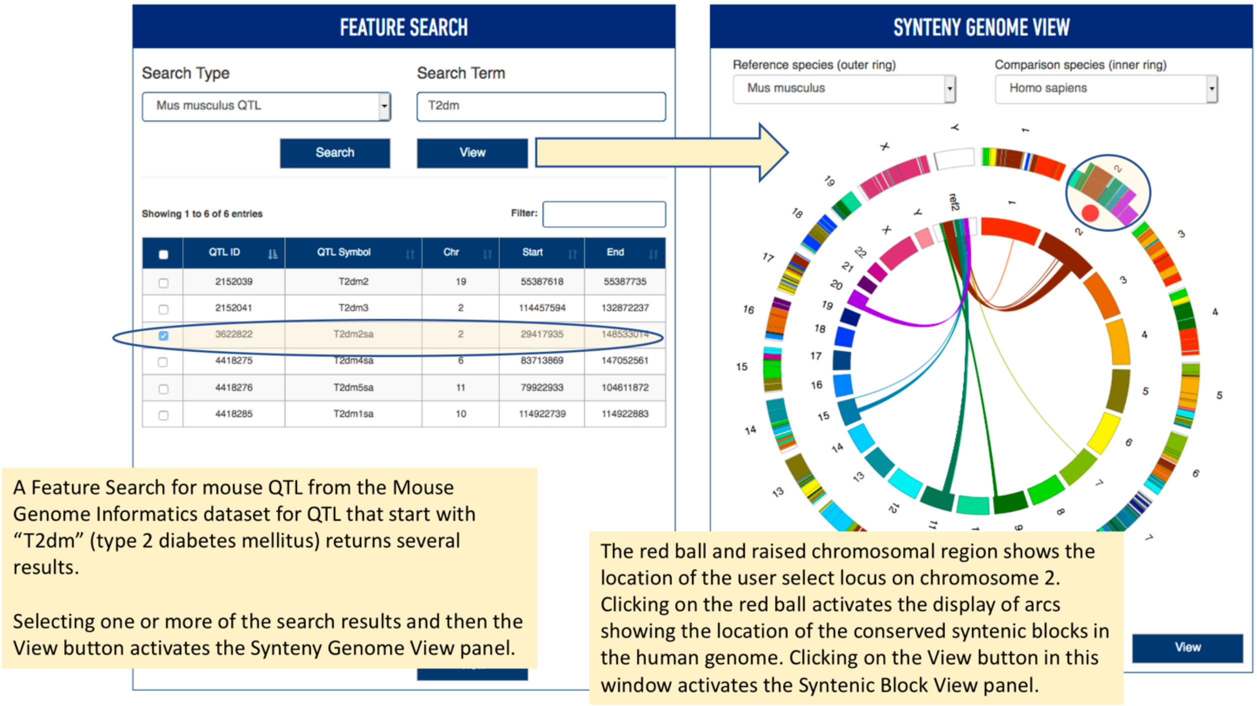

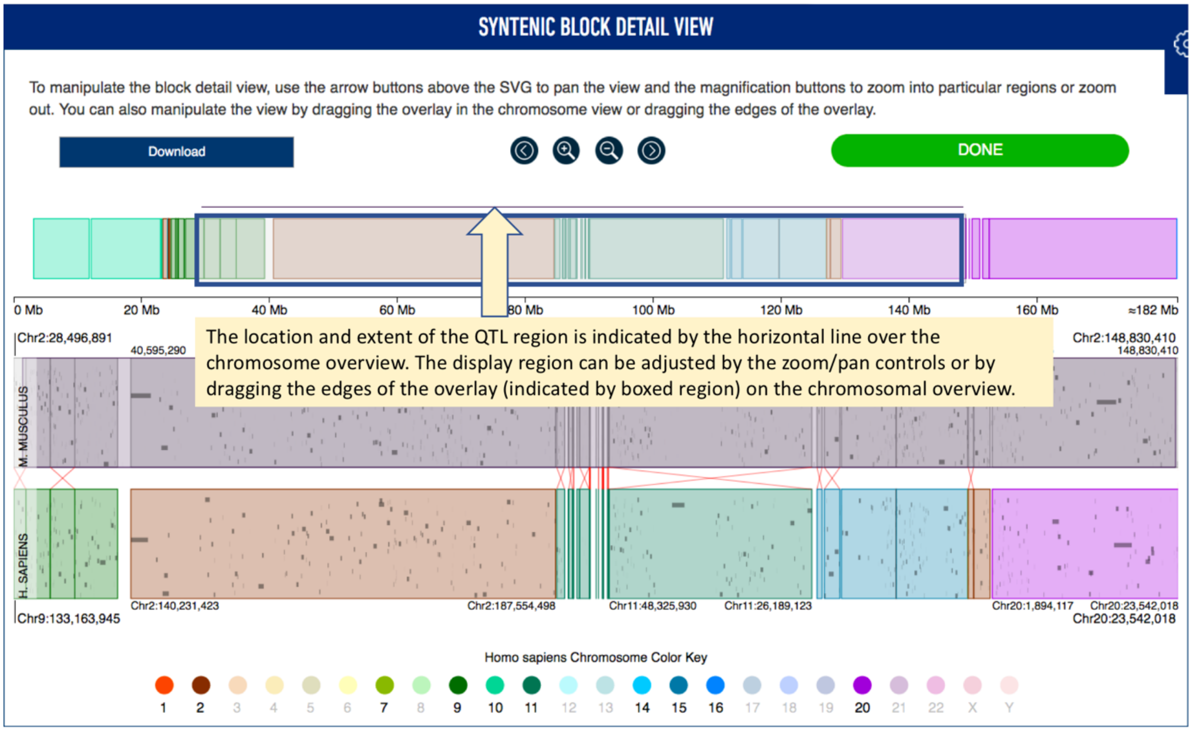

To explore annotated functions and phenotype associations of mouse genes within and around the QTL region the researcher could limit the annotation searches to the mouse (Reference) genome and then use the **Syntenic Block Features Display Filters** tool to find genes annotated to relevant phenotype terms from the Mammalian Phenotype (MP) ontology (Smith and Eppig 2012). A search for the MP term, impaired glucose tolerance, identifies thirteen genes that fall within the boundaries of the *T2dm2sa* QTL interval: *Pkn3, Lcn2, Dpm2, Zbtb43, Bbs5, Commd9, Hipk3, Pax6, Hdc, Ap4e1,Chgb, and Pcsk2*. Search results are automatically displayed in the **Syntenic Block Detail View** panel.

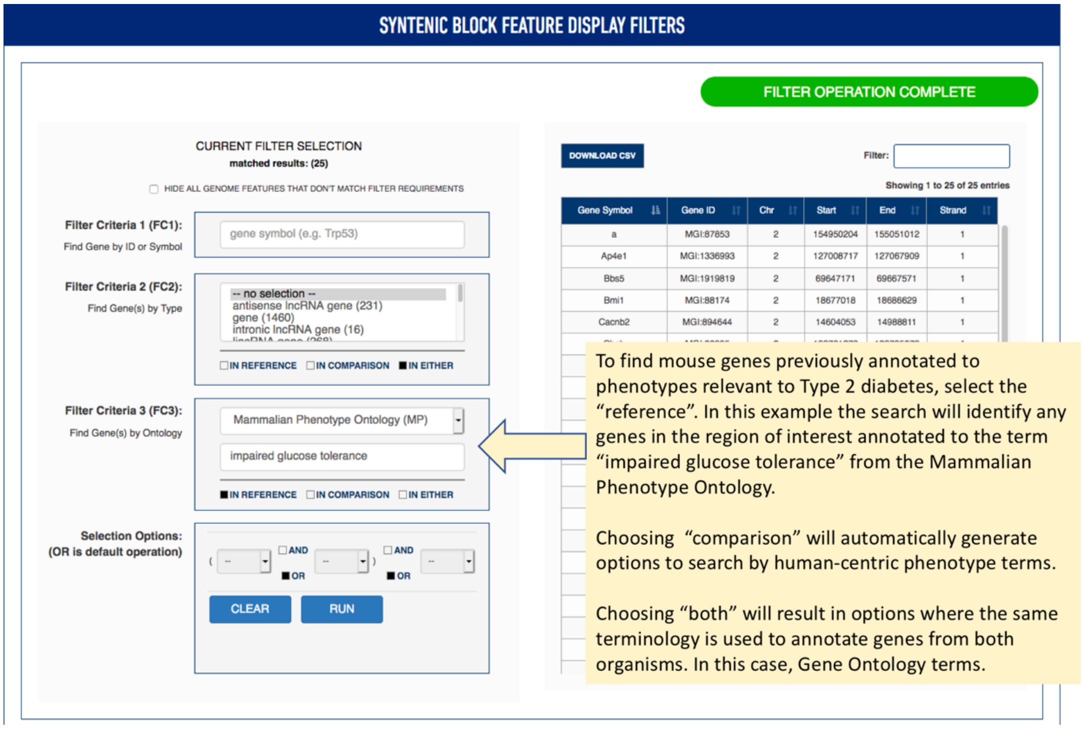

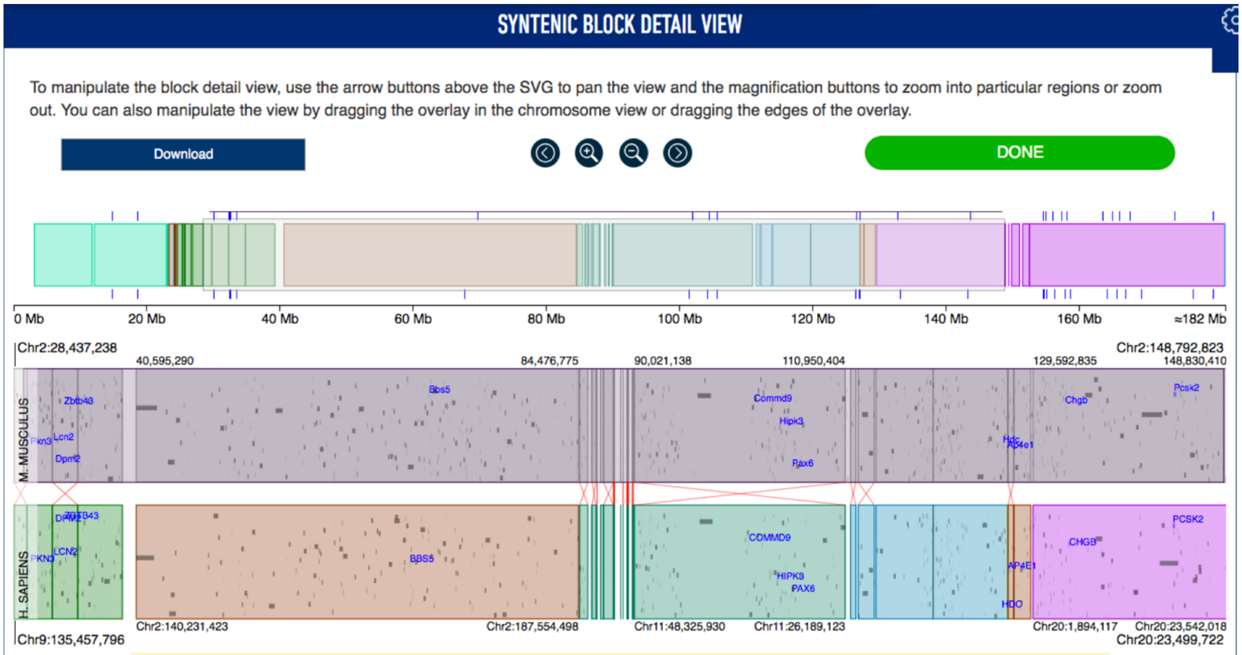

Results of the display filter search output is automatically displayed in the Syntenic Block Detail View. Hash marks along the chromosome overview show the location of the matching genome features. The orthologs in the other genome are also displayed.
